# Supplementary material for: Dual-Hit Model of Parkinson’s Disease: Impact of Dysbiosis on 6-Hydroxydopamine-Insulted Mice—Neuroprotective and Anti-Inflammatory Effects of Butyrate
Source: Int J Mol Sci. 2022 Jun 7;23(12):6367. doi: 10.3390/ijms23126367 (PMC9223521; doi:10.3390/ijms23126367)
Supplement: Supplementary file 1 [file ijms-23-06367-s001.zip › ijms-1727819-supplementary material.pdf]

Figure S1

A

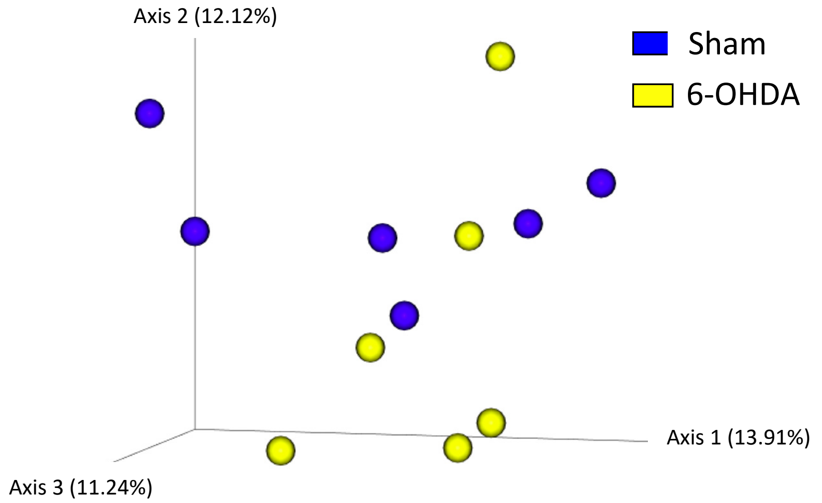

ANOSIM results: R=0.189 (p= 0.042)

B

**Key genera**<br>• **Key species (n°ASVs)**	**Sham**	**6-OHDA**	**p-value**	**LDA score**
U.g.of Prevotellaceae (4)	0	0.42 ± 0.16	0.022	2.22
• *Prevotellamassilia timonensis* (3)	0	0.42 ± 0.16	0.022	5.21
U.g. of Lactobacillaceae (17)	0.94 ± 0.39	0.40 ± 0.24	0.076	–
• *Ligilactobacillus* (11)	0.87 ± 0.32	0.33 ± 0.17	0.006	5.87
U.g. of Clostridia (7)	0	0.18 ± 0.08	0.007	2.11
• *U.s. of Eubacteriales* (5)	0	0.16 ± 0.05	0.007	5.12
U.g. of Enterobacteriaceae (15)	0	0.03 ± 0.01	0.058	–
U.g. of F16 (17)	0.31 ± 0.24	0.85 ± 0.30	0.076	–

Figure S1. Key features of 6-OHDA mice gut microbiota. A) Unweighted UniFrac-based 3D PCoA plot of 6-OHDA and Sham fecal communities. Analysis of similarity (ANOSIM) with 999 permutations was used to detect the statistical significant differences in microbial community composition between groups; on the bottom of plots are reported both R statistics and p values. B) Table showing key genera discriminating 6-OHDA and Sham mice and their key species according to BLAST taxonomy classification. The key features were identified based on linear discriminant

analysis (LDA) combined with effect size (LEfSe) algorithm (alpha values of 0.05 for both Kruskal–Wallis and pairwise Wilcoxon tests and a cutoff value of LDA score (log10) above 2.0). In table means and STE, p-values and LDA-scores for each key phylotype are shown.

Figure S2

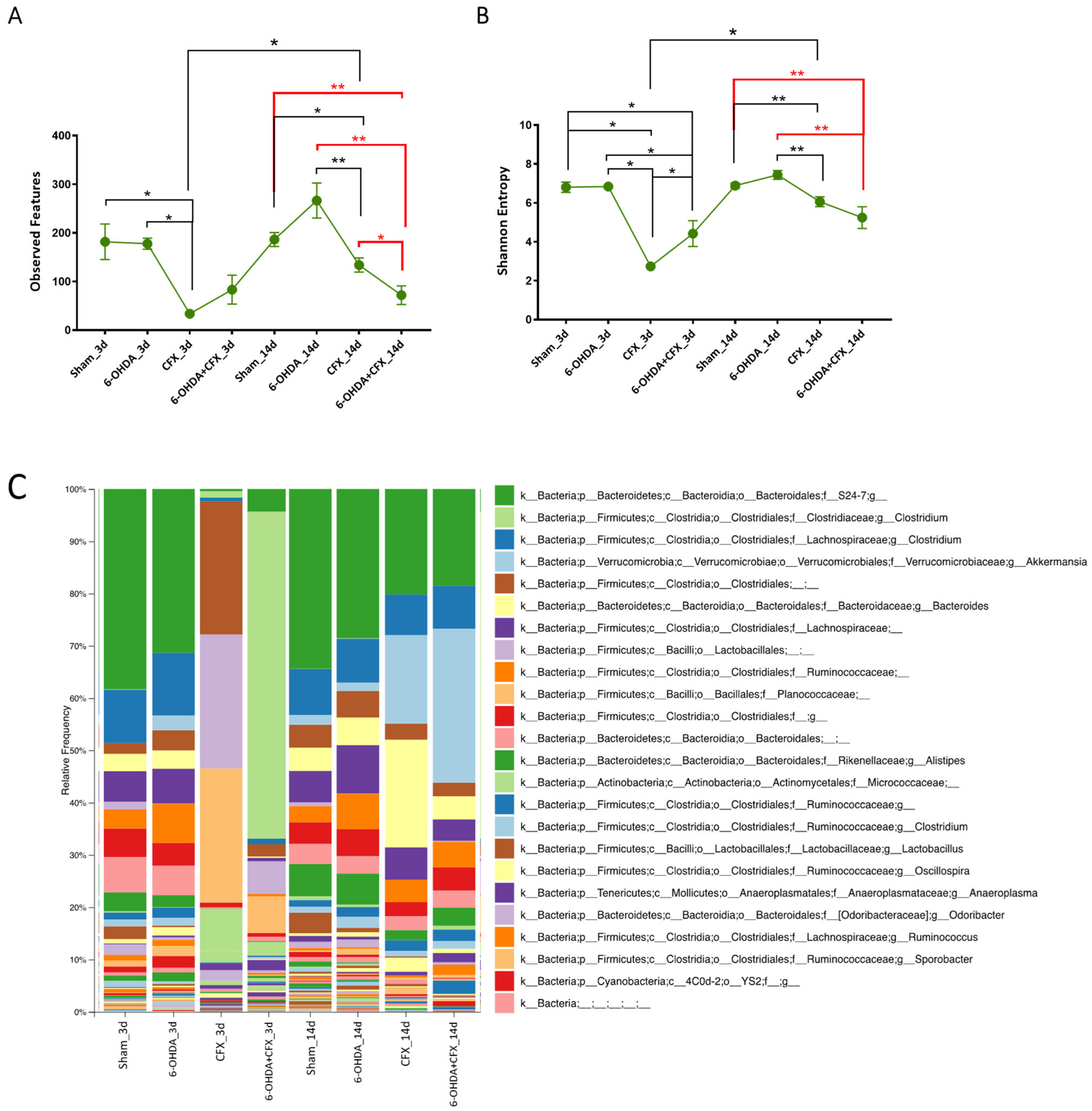

Figure S2. Analysis of microbial communities assortment at 3 and 14 days from antibiotic treatment. A-B) Results of Observed Features and Shannon Entropy presented as mean ± SEM. Asterisks indicate a significant difference as obtained by pairwise Kurskal-Wallis test (*$p < 0.05$, **$p < 0.01$). C) Bar chart showing the mean relative abundance of all bacterial ASVs taxonomically classified at genus level; the legend shows the top 24 bacterial genera among the whole proportion.
